# Absence of Behavioral Harm Following Non-efficacious Sexual Orientation Change Efforts: A Retrospective Study of United States Sexual Minority Adults, 2016–2018

**DOI:** 10.3389/fpsyg.2022.823647

**Published:** 2022-02-02

**Authors:** D. Paul Sullins

**Affiliations:** ^1^Department of Sociology, The Catholic University of America, Washington, DC, United States; ^2^The Ruth Institute, Lake Charles, LA, United States

**Keywords:** sexual orientation change efforts, conversion therapy, minority stress, suicide, sexual orientation

## Abstract

**Background:**

Do sexual minority persons who have undergone unsuccessful sexual orientation change efforts (SOCE) suffer subsequent psychological or social harm from the attempt? Previous studies have conflated present and past, even pre-SOCE, harm in addressing this question. This study attempts, for the first time, to isolate and examine the question of current psychosocial harm for former SOCE participants among sexual minorities in representative population data.

**Method:**

Using nationally representative data (*n* = 1,518) across three cohorts of sexual minorities (centered in 1969, 1987, and 2003) in the United States (U.S.), persons exposed to SOCE were compared with the remainder not exposed to SOCE on two measures of internal distress—psychological distress (Kessler scale) and current mental health—and seven measures of behavioral harm: substance abuse (DUDIT); alcohol dependence (AUDIT-C); self-harm; suicide ideation; suicide planning; suicide intentions; and suicide attempts.

**Results:**

The SOCE group was statistically indistinguishable from the non-SOCE group on any measure of harm. For behavioral harm, risk ratios were 0.97–1.02. Harm was equivalent for the two groups despite the SOCE group having experienced higher lifetime and current minority stress, greater childhood adversity, and lower socioeconomic status. Logistic regression models that adjusted for these differences suggest that SOCE exposure reduced the effect of minority stress and childhood adversity for suicide attempts, although this effect did not fully account for the equivalence between the SOCE and non-SOCE groups.

**Conclusion:**

Despite higher exposure to factors predicting behavioral harm—minority stress, childhood adversity, and lower socioeconomic background—sexual minority persons who had undergone failed SOCE therapy did not suffer higher psychological or social harm. Concerns to restrict or ban SOCE due to elevated harm are unfounded. Further study is needed to clarify the reasons for the absence of harm from SOCE.

## Background

Sexual orientation change efforts (SOCE) is a summary term for therapies or programs that support change from same-sex to opposite-sex orientation in sexual attraction, identity, and/or behavior. The practice is subject to intense controversy. Sharply divergent bodies of research have presented evidence that it is ineffective and harmful (Drescher et al., 2016, p. 7; Blosnich et al., 2020; Ryan et al., 2020) and that it is effective and of psychological benefit (Beckstead and Morrow, 2004; Jones and Yarhouse, 2011; Dehlin et al., 2015; Sullins et al., 2021). Currently, 20 U.S. states have imposed limited restrictions on SOCE, while in four states injunctions or legislation prohibit such bans (Movement Advancement Project, 2020). Opponents to SOCE claim, and proponents deny, that it currently includes proscribed techniques, such as aversive punishment or electric shock (Flentje et al., 2013; Rosik, 2017), and bans are typically written very broadly. The American Psychological Association (APA) Task Force on the topic has expressed concern that the practice of SOCE has “become mired in ideological disputes and competing political agendas” (American Psychological Association, Task Force on Appropriate Therapeutic Responses to Sexual Orientation, 2009, p. 92).

The differing findings may be due, in part, to differing definitions regarding what constitutes SOCE. The precise range, harshness and focus of SOCE interventions is in dispute, with opponents defining them more broadly, aversively, and focused on same-sex conversion, while proponents define them as benign talk therapy or teaching that may or may not include changing sexual orientation in accord with the client’s goals.

As recently argued elsewhere (Sullins et al., 2021), the disparate research findings on SOCE may be due to incommensurable samples selected for study: those who find little efficacy but substantial harm from SOCE tend to select samples “exclusively or mostly dominated by LGB (lesbian, gay, or bisexual) identified participants” (Sullins et al., 2021), that is, persons for whom SOCE has, by definition, failed with respect to sexual orientation identity; while those who find more efficacy but little harm tend to select samples largely from persons who reject a former LGB identity (Jones and Yarhouse, 2011; Pela and Sutton, 2021; Sullins et al., 2021), that is, persons for whom SOCE has by definition succeeded in reframing sexual orientation identity. Two recent studies have examined samples comprised of both types of former SOCE participants, both finding no difference in harm (Lefevor et al., 2020; Rosik et al., 2021).

Recent claims of harm from unsuccessful SOCE, that is, among former SOCE participants among current sexual minority persons, have also entailed another form of bias, arguing from a lifetime association of increased psychological morbidity with SOCE exposure that SOCE induces harm (Meanley et al., 2020; Ryan et al., 2020; Salway et al., 2020; del Río-González et al., 2021). The analytical challenge for such claims is that, unlike the morbidity observed, SOCE therapy is not lifelong or continuous, but is confined to a definite, restricted time in the life course, usually lasting less than a year. Recent re-examination of one such study by Blosnich et al. (2020, 2021) claiming that SOCE “may compound or create … suicidal ideation and suicide attempts” has found that failure to adjust for pre-existing suicidality invalidates its conclusions (Blosnich et al., 2020; Sullins, 2021). The problem was not only that much of the harm predated SOCE exposure and that harm following SOCE was substantially reduced compared to sexual minorities who had never engaged in SOCE, but also that other precipitating factors that may account for harm were not examined.

Blosnich et al. (2020), using the same data as the present study, did improve upon previous research by controlling for demographic covariates (although as we shall see their set of measures can be improved), and they carefully considered the differential experience of adverse childhood experiences (ACEs). However, they ignored every other factor that may have affected differences in outcome associated with SOCE exposure. They did not consider any comorbidities, such as alcohol dependence, substance abuse, self-harming behavior, mental health, or psychological distress, all of which are known to be associated with suicidal morbidity and were measured in the Generations data used in their study. While speculating about the association of concealment with SOCE, they did not consult the well-constructed measure of concealment present in their own study’s data, which contradicted their speculation (as discussed further below). Most notably, although Blosnich et al. (2020, 2021) included strong proponents of minority stress theory (MST), which attributes harm outcomes among sexual minorities to the effect of stigma, discrimination, and other social stress, and despite speculating that higher stress may explain higher suicidality among former SOCE participants, they did not examine any of the multiple indicators of minority stress that were readily available in the data.

The aim of the present study is to amend this gap in the evidence, in part, by examining a larger set of covariates that may (or may not) help explain observed differences in harm outcomes for sexual minority persons who have been exposed to SOCE. Such an analysis, as already noted, has limited application to the adjudication of SOCE harm in that it does not represent all SOCE alumni, but only those who have not succeeded in at least one of the possible aims of SOCE, i.e., to support the re-orientation of persons from sexual minority to heterosexual identity. This limitation notwithstanding, comparison of this group with sexual minority persons not exposed to SOCE can shed light on two theoretical questions pertaining to the question of harm from SOCE. First, how much harm actually exists? Since studies of unsuccessful SOCE participants have generally agreed in finding higher harm than among successful ones, examining the unsuccessful SOCE participants can help determine the upper extent of possible harm. Recognizing the amount of harm among current sexual minority persons, moreover, is of clinical significance for this population even if net harm from SOCE, when both successful and unsuccessful participants are considered, were negative. As noted, prior studies finding harm from SOCE in the sexual minority population have only examined lifetime harm, including harm that occurred before SOCE, not present harm. The present study, by contrast, aims to examine the possibility of present harm associated only with past SOCE involvement, not possible future SOCE. This is important for the question of causation, since harm associated with possible future SOCE cannot be an effect of SOCE involvement. Second, to what extent, if any, is current harm associated with past SOCE affected or explained by early life covariates, current comorbidities, or differences in minority stress? To the extent that both SOCE and harm associated with SOCE are mutually associated with factors that may independently precipitate harm, the attribution of such harm to SOCE involvement is spurious. In this case, SOCE involvement would serve merely as a marker for a set of conditions that would predict harm for any sexual minority person, regardless of SOCE involvement. The answers to these questions may help to characterize the sources of SOCE-linked harm, or its absence, with broad application to theories of sexual minority psychological distress and clinical interventions and have particular application to the controversial question whether SOCE therapy should be banned to prevent harm to sexual minority persons. To my knowledge, the present study is the first empirical examination of such questions in population-representative data.

## Data

The data for this study were collected as part of the Williams Institute’s Generations study, an epidemiological study designed to examine the health and wellbeing of three generational cohorts of non-transgender sexual minority persons in the United States (Meyer, 2020). The cohorts consisted of persons aged 52–59 (Pride Generation), whose sexual coming of age took place around the time of the Stonewall riots and the start of the gay liberation movement; persons aged 32–41 years (Visibility Generation), whose early life experiences coincided with the beginning of the AIDS (acquired immune deficiency syndrome) epidemic and greater visibility and social acceptance for LGB people; and those aged 18–25 in 2016 (Equality Generation), whose early life experiences were affected by the growing focus on LGB marriage and employment equality. Eligibility was also restricted to the three largest United States racial and ethnic groups (Black, Latino, or White, although multi-ethnic identities that included one of these were also included; Krueger et al., 2020).

Participants were screened by the Gallup Organization’s daily random digit dialing assay (both landline and cell phones) for 1 year beginning March 2016. Recruitment for Black and Latino participants extended for an additional year, until March 2018. Respondents who identified as “lesbian, gay, or bisexual” but not transgender (who were recruited into a separate companion study) were invited to complete a self-administered online or paper questionnaire, which required fifth-grade English proficiency. Calls to 366,640 Americans resulted in a sample of 3,525 eligible participants (1%), of which 1,518 (43%) completed usable interviews. Statistical weighting adjusted for the complex survey sample design, differential non-response, the extended sample of Black and Latino respondents, and known characteristics of the sexual minority population as reflected in prior data collected by Gallup and the United States Census. The resulting data are designed to be generalizable to the United States population of sexual minority adults and have formed the basis for numerous prior studies and estimates for this population (Nock et al., 2009; Rothblum et al., 2020; Meyer et al., 2021). More information about the study’s methodology and sample characteristics is available online at http://www.generations-study.com and in several published reviews and methodological reports (Krueger et al., 2020; Meyer, 2020; Meyer et al., 2020).

## Measures

For SOCE participation respondents were asked, “Did you ever receive treatment from someone who tried to change your sexual orientation?” and if so, their age when such treatment last occurred. Sociodemographic covariates included race and ethnicity, with categories of white, black, Hispanic, and other; educational attainment, with categories of high school or less, some college, college degree, and more than a college degree; poverty income, ranging from below poverty to over four times poverty income; self-rated current health, with categories of excellent, very good, good, fair, and poor; sexual identity, with categories of lesbian, gay, bisexual, queer, pansexual, asexual, and other; sex assigned at birth, with categories of man and woman; and age in years.

### Minority Stressors

The Generations data contained extensive measures of minority stress. Seven variables in all captured indicators of discrimination, stigma, or other stressors that could be related to SOCE. In addition to lifetime discrimination and childhood bully victimization, two variables—everyday discrimination and stressful life events—assessed current discrimination experiences. Follow-up questions for these four variables enabled the respondent to specify adverse experiences due to minority sexual orientation. Two variables—felt stigma and internalized homophobia—measured the respondents’ attitudes, or perceptions of others’ attitudes, due to their LGB identity. Finally, a series of variables labeled “chronic strains” assessed personal stress conditions, such as isolation, exhaustion, or conflict, but not specifically due to sexual orientation or gender identity (SOGI). The following paragraphs describe these variables in more detail.

#### Discrimination Since Age 18

A series of nine items asked respondents how often since age 18 they had experienced discriminatory behavior or violence, such as being physically attacked or sexually assaulted, had property stolen or vandalized, had been verbally insulted, or had been denied a job or promotion. The response options for the nine items were never, once, twice, and three or more times. Analyses incorporating multiple instances were explored and found to differ very little from those simply reflecting the extent of any discrimination, so the last three response options were collapsed to form indicator variables indicating any experience (vs. none) of each of the nine forms of discrimination. One item, on housing discrimination, yielded too few cases of sexual orientation discrimination (only 12) and was dropped from the analysis. The remaining eight indicators were then combined into a summative scale ranging from zero to nine expressing the extent of discrimination experienced by each respondent.

#### Stressful Life Events or Perceived Stress in the Past 12 Months

Eleven items asked about adverse experiences in the past 12 months. Most of these were similar to the events in the lifetime discrimination sequence, for example being robbed, having property destroyed, or fired from a job. Some items were very different, however, such as getting divorced or suffering a major financial crisis. Response options were only yes or no, indicating whether the event had occurred or not.

#### Everyday Discrimination in the Past Year

Another series of nine items asked whether “in your day-to-day life over the past year” the respondent had experienced such less extreme adverse actions as being treated with less courtesy or respect than other people, receiving poorer service at restaurants, being threatened or insulted, or having people act as if they were not honest or smart, or felt superior or were afraid of them. Response options were often, sometimes, rarely, and never. A variable constructed by the Generations staff reversed and combined the nine items into a single variable expressing the respondent’s current level of everyday discrimination experience ranging from 1 (low) to 4 (high).

#### Childhood Bully Victimization

Respondents were asked “How often, if ever, were you bullied before you were 18 years old?” The mean of the 4-point response scale (often, sometimes, rarely, and never) was reverse coded so that higher scores indicated more frequent childhood bully victimization.

For the previous four measures, respondents who indicated having experienced any of referenced discriminatory behavior were asked follow-up questions whether the adverse experiences had been due to their age, sex, sexual orientation, transgender status, gender presentation, race/ethnicity, physical appearance (e.g., weight, height) or religion/spirituality. Respondents were instructed to indicate all causes that applied. Persons who included sexual orientation, transgender status, and/or gender presentation among the causes they listed were classified as having received SOGI discrimination, regardless of what other causes they may have also indicated. Those who did not include one or more of these three causes were classified as having received non-SOGI discrimination. SOGI discrimination and non-SOGI discrimination were therefore not mutually exclusive; a respondent could report experiencing multiple or intersecting forms of discrimination for the same or different adverse experiences.

#### Felt Stigma

Three items asked the respondent’s level of agreement or disagreement with statements about the perceived opinion of “most people (employers) where I live”: they “think less of a person who is LGB”; “will hire openly LGB people if they are qualified for the job”; and “would not want someone who is openly LGB to take care of their children.” Response options were strongly disagree, somewhat disagree, neither agree nor disagree, somewhat agree, and strongly agree. A constructed variable included on the data file combined these items, the second one reverse coded, into a measure of current stigmatizing attitudes in the respondent’s community of residence.

#### Unconcealment (“Out”)

Four questions asked whether the respondent had disclosed his or her sexual identity to all, most, some or none of his or her family, co-workers, heterosexual friends or health care providers. Alpha (the intercorrelation coefficient) for the four items was 0.80. Following Pachankis and Bränström (2019), the degree of unconcealment or “outness” was measured as the average of those responding over the four items, thereby expressing the proportion of sexual identity disclosure to the groups to which one could reveal one’s sexual identity.

### Other Stressors

Other items measured stress that was not specifically identified as being related to minority sexual identity. This is not to imply that these stressors may not be empirically associated with sexual minority status, but only that they were not measured as pertaining uniquely to sexual minorities.

#### Adverse Childhood Experiences

The ACE score was expressed as the additive index of eight indicators of childhood experiences identified by the Centers for Disease Control and Prevention (CDC) to be negatively related to adult health outcomes: sexual abuse; physical abuse; emotional abuse; substance abuse in the household; intimate partner violence in the household; mental illness in the household; a family member imprisoned; and parental separation or divorce. Three ACEs figured prominently in the analysis. Mental illness, emotional abuse, and sexual abuse were assessed with questions asking whether before age 18 the respondents had “live[d] with anyone who was depressed, mentally ill, or suicidal,” had been sworn at, insulted or put down, or had been made to have sex or touch or be touched sexually by someone at least 5 years older. Indicator variables coded 1 for the presence or zero for the absence of each of these experiences.

#### Chronic Strains

Respondents rated 12 statements about their current lives as true, somewhat true or not true. The items related to general personal difficulties in life management, such as “you are trying to take on too many things at once,” “your job often leaves you feeling both mentally and physically tired,” “and you are alone too much.” A three-point scale of general chronic strains was aggregated from the responses. Unlike the other stressors measured, chronic strains were not specifically related to minority sexual orientation.

#### Internalized Homophobia

Internalized homophobia was a measured by agreement or disagreement five statements relating to dissatisfaction with having an LGB identity. Response options were strongly disagree, somewhat disagree, neither agree nor disagree, somewhat agree, and strongly agree. Two of the component items were collinear with SOCE therapy: “I have tried to stop being attracted to people who are the same sex as me;” and “I would like to get professional help in order to change my orientation from LGB to straight.” These two items, which report on past or prospective actions, were not highly intercorrelated with the remaining three items on the scale, all of which report current attitudes: “If someone offered me the chance to be completely heterosexual, I would accept the chance”; “I feel that being LGB is a personal shortcoming for me;” and “I wish I were not LGB.” Removing the first two items improved scale alpha from 0.75 to 0.83. Thus, to achieve an unbiased measure for purposes of the present study, the scale was adjusted to include only the last three items. Average response on the items were combined to create a scale ranging from 1 to 5 measuring internalized homophobia.

#### Psychological Distress (Kessler Scale)

The Generations survey included the Kessler Scale of Psychological Distress (K6), consisting of six questions designed “to identify persons with a high likelihood of having a diagnosable mental illness and associated functional limitations” (Pratt et al., 2007). This 24-point scale, developed by a Harvard Medical School team led by Dr. Ronald Kessler (Kessler et al., 2003), has been validated by dozens of studies and is used to estimate the prevalence of mental illness in WHO surveys worldwide as well as most National Health Surveys in the developed world, including those of Germany, Australia, Canada, and the United States. Following Kessler’s scoring scheme and CDC usage, persons scoring 13 or higher were classified as experiencing non-specific serious psychological distress (SPD).

#### Current Negative Mental Health

Respondents were asked, “Now thinking about your mental health, which includes stress, depression, and problems with emotions, for how many days during the past 30 days was your mental health not good?” The reported number of days was taken as an indicator of the current level of negative or poor mental health.

### Behavioral Harm

#### Alcohol and Substance Abuse

Alcohol and substance abuse were measured using the Alcohol Use Disorder Identification Test (AUDIT-C; World Health Organization, 2001) a 3-item scale ranging from 0 to 12, and the Drug Use Disorders Identification Test (DUDIT), (Berman et al., 2005) an 11-item scale ranging from 0 to 35. Both instruments are responsive to criteria of the American Psychiatric Association’s (APA’s) Diagnostic and Statistical Manual (DSM), widely used internationally, and have been well-validated for good psychometric properties in predicting active alcohol or substance use disorders (Berner et al., 2007; Hildebrand, 2015). Following prior research (Berman et al., 2005; Berner et al., 2007), and as recommended by the Generations data documentation (Krueger et al., 2020), the prediction cutoff score for alcohol-related disorder on the AUDIT-C was assessed at 5 for men and 4 for women, and the score for substance-related disorder on the DUDIT was set at 6 for men and 2 for women.

#### Suicidal Behavior

Suicidal behavior was assessed using an instrument developed by the Unite States Army to assess risk in service members (Nock et al., 2014). Four questions asked, “Did you ever … in your life have thoughts of killing yourself?”; … have any intention to act on thoughts of wishing you were dead or trying to kill yourself?; … think about how you might kill yourself (e.g., taking pills, shooting yourself) or work out a plan of how to kill yourself?; … make a suicide attempt (i.e., purposefully hurt yourself with at least some intention to die)?” Response options were “No,” “Yes, once,” and “Yes, more than once.” Follow-up questions for the yes responses asked how old the respondent was when they engaged in the suicide behavior or both the first and most recent of multiple instances of that behavior. For each behavior, current suicidality was indicated by having engaged in the behavior at least once in the past year, as indicated at Wave 1 or Wave 2.

#### Self-Harm

The survey instrument also asked about purposeful non-suicidal harm to oneself, such as “cutting yourself, hitting yourself, or burning yourself.” As with suicidal behavior, follow-up questions specified the timing and single or multiple instances. Current self-harming behavior was measured by a variable indicating the presence of any self-harming behavior in the past year.

## Materials and Methods

The analysis proceeded in two stages corresponding to the two analytical questions of interest. First, the difference of means or proportions between the SOCE and non-SOCE groups for behavioral harm outcomes, covariates, and stressors was examined to determine the presence and extent of harm from SOCE. This analysis was performed using unadjusted population-weighted estimates so as to accurately reflect the level of harm in the presenting clinical population. Statistically significant differences were assessed by *t* test or *f* test as appropriate and effect sizes computed for those differences that were significant. In the second stage, possible covariate influence on harm associated with SOCE was examined by assessing the odds ratio for SOCE therapy from logistic regression models predicting each behavioral harm outcome after adjustment for demographic characteristics, early life covariates, current comorbidities, and differences in minority stress.

All analyses were adjusted for the complex sample design and employed survey weights to allow for generalization to the United States population of sexual minority adults, ages 18–27, 32–43, and 50–61. All regression models were certified for proper model specification using the Pregibon/Tukey goodness of link test (Pregibon, 1980) and for acceptable fit to the data using the Hosmer and Lemeshow goodness of fit procedure for complex sample designs (Archer et al., 2007). The variance inflation factor (VIF) for all included covariates ranged from 1.1 to 2.75, indicating a low level of multicollinearity. Analyses were performed using SPSS 25 and Stata 13 statistical software. As a secondary analysis of pre-existing public data, the present study’s methods were determined to be exempt from human subject ethical review under 45 CFR 46.104 by the Catholic University of America Institutional Review Board in Certificate 21-0016 issued March 12, 2021.

## Results

### Sample Characteristics

Table 1 presents selected demographic characteristics of the sample, comparing sexual minority persons with SOCE experience (“SOCE alumni”) with those who had not experienced SOCE.

**Table 1:** Demographic characteristics of lesbian/gay individuals, by experiencing sexual orientation change efforts (SOCE), counts, and weighted proportions: probability sample of sexual minorities, United States, 2016–2018 (*N* = 1,518).

	Overall sample, *N* (%; SE) or mean (SE)	Experienced SOCE		*P*
		NO *N* (%; SE) or mean (SE)	YES *N* (%; SE) or mean (SE)	
SOCE experience		1,410 (93.1; 0.81)	108 (6.9; 0.81)	–
**Income (as percent of poverty income)**				
<100%	210 (19.1; 1.37)	187 (18.4; 1.41)	23 (29.3; 5.71)	0.0631
100–199%	279 (22.5; 1.41)	251 (22.0; 1.45)	28 (29.9; 5.86)	0.1912
200–299%	192 (12.7; 1.06)	179 (12.8; 1.10)	13 (11.6; 3.55)	0.7468
300%+	810 (45.6; 1.60)	766 (46.9; 1.66)	44 (29.3; 5.35)	0.0017
**Educational attainment**				
High school diploma or less	309 (42.5; 1.69)	283 (41.9; 1.75)	26 (50.9; 6.08)	0.1554
Some college	492 (31.9; 1.40)	464 (32.4; 1.46)	28 (25.6; 4.79)	0.1767
College degree	429 (16.0; 0.86)	403 (16.4; 0.91)	26 (10.4; 2.27)	0.0152
More than a college degree	288 (9.6; 0.62)	260 (9.3; 0.64)	28 (13.1; 2.77)	0.1913
Age in years	30.9 (0.37)	30.7 (0.38)	32.7 (1.43)	0.1846
**Sexual identity**				
Lesbian/Gay	833 (46.9; 1.59)	757 (45.3; 1.64)	76 (68.0; 5.94)	0.0002
Bisexual	493 (40.6; 1.62)	476 (42.0; 1.68)	17 (21.7; 5.70)	0.0006
Other sexual identity	181 (12.5; 1.04)	166 (12.7; 1.09)	15 (10.3; 3.14)	0.0000
**Race/Ethnicity**				
White	931 (59.5; 1.56)	871 (60.2; 1.61)	60 (50.5; 6.14)	0.1284
Black	180 (13.5; 1.09)	162 (12.8; 1.10)	18 (22.2; 5.36)	0.0858
Latino	158 (10.8; 0.98)	145 (10.7; 1.01)	13 (13.1; 4.10)	0.5696
Other racial/ethnic identity	249 (16.2; 1.14)	232 (16.3; 1.18)	17 (14.2; 4.21)	0.6234
**Health**				
Poor	56 (3.7; 0.62)	49 (3.5; 0.63)	7 (6.7; 2.94)	0.2859
Fair	206 (16.2; 1.27)	189 (16.1; 1.31)	17 (17.6; 5.10)	0.7809
Good	480 (33.2; 1.53)	448 (33.6; 1.59)	32 (27.7; 5.33)	0.2942
Very good	569 (34.9; 1.51)	538 (35.2; 1.56)	31 (30.0; 5.75)	0.3821
Excellent	187 (12.0; 1.04)	167 (11.6; 1.06)	20 (18.0; 4.52)	0.1701
**Sex at birth**				
Woman	812 (60.0; 1.53)	765 (60.8; 1.57)	47 (49.2; 6.15)	0.0679
Man	706 (40.0; 1.53)	645 (39.2; 1.57)	61 (50.8; 6.15)	0.0679

*Values shown are weighted for population and survey design. CI, confidence interval; RR, risk ratio*.

Blosnich et al. (2020, 2021) present the same findings for education, age, sexual identity, and race, but do not include income and health status. Blosnich et al. (2020, 2021) reported gender identity (with categories of man, woman, and non-binary) rather than sex assigned at birth (with categories of male and female), however in the present analysis breaking out the small number (94) of binary cases introduced an unacceptable number of empty cells, and since the Generations sample screened out transgender persons, all persons identifying as man or woman were congruent with their birth sex, so the two measures are the same for these categories. Table 1 reports raw case numbers, but population-weighted percentages within covariates, to facilitate comparing the SOCE and non-SOCE groups.

As Table 1 shows, 6.9% of sexual minority persons in the United States have experienced SOCE therapy of some sort. This group differed significantly from the remainder who had not undergone SOCE (93.1%) on several demographic dimensions. SOCE alumni tended to be less affluent, less educated, less white, more black, and more male than were the persons who had not undergone SOCE. A total of 29% of the SOCE alumni were in poverty, compared to only 18% of the non-SOCE group; and almost half (47%) of the non-SOCE group, compared to under a third (29%) of the SOCE group, had income greater than three times the poverty level. Over half (51%) of the SOCE group, compared to only 42% of the non-SOCE group, had only a high school diploma or less education, and smaller proportions of the SOCE group had begun or finished college. Table 1 likely understates these educational differences relative to the actual population, since sample selection screened out persons with less than a fifth-grade education. The SOCE alumni included a higher concentration of black persons and a lower concentration of women than the non-SOCE group; these differences were significant at the 0.10 critical level. SOCE alumni were no less healthy than the non-SOCE group, although their health was a little more diverse; higher proportions of the SOCE alumni reported being both in excellent and in poor health. Over a fifth (22%) of SOCE alumni were black, and although three-fifths (60%) of sexual minorities were female, the SOCE group was evenly split between men and women.

Table 2 compares the SOCE and non-SOCE groups on psychological health and the experience of minority stress. According to the MST, higher exposure to minority stressors should be related to lower psychological health, but in these data the SOCE alumni experienced higher trauma and discrimination, but did not express higher psychological distress, than did sexual minorities who had not experienced SOCE.

**Table 2:** Minority stressors and mental health by experiencing SOCE, counts, and weighted proportions: probability sample of sexual minorities, United States, 2016–2018 (*N* = 1,518).

	Overall sample (*n* = 1,518), %; SE or mean; SE	Experienced SOCE		*P*	Effect size (*d*)
		No (*n* = 1,410), %; SE or mean; SE	Yes (*n* = 108), %; SE or mean (SE)		
**Stressors**					
Adverse childhood experiences (ACEs)—mean (0–8)	3.33; 0.071	3.27; 0.073	4.18; 0.306	0.0041	0.035
Bullied in high school due to sexual orientation or gender presentation—mean (0–4)	1.89; 0.041	1.85; 0.041	2.50; 0.169	0.0002	0.478
Percent often bullied in high school due to sexual orientation or gender presentation	19.1; 1.29	17.6; 1.29	38.0; 6.08	0.0011	0.485
Lifetime victimization/discrimination due to sexual orientation or gender presentation—mean (0–8)	1.44; 0.071	1.29; 0.067	3.47; 0.379	0.0000	0.844
Percent ever assaulted due to sexual orientation or gender presentation	18.7; 1.24	16.8; 1.23	43.8; 6.12	0.0000	0.626
Everyday discrimination (past year) due to sexual orientation or gender presentation mean (0–4)	1.51; 0.027	1.48; 0.027	1.92; 0.125	0.0005	0.641
Felt stigma (past year)—mean (0–8)	2.71; 0.030	2.68; 0.031	3.13; 0.097	0.0000	0.338
Chronic strains (past year)—mean (0–8)	1.62; 0.011	1.61; 0.012	1.74; 0.050	0.0103	0.397
Internalized homophobia (adjusted)—mean (1–5)	1.63; 0.029	1.62; 0.030	1.81; 0.114	0.1062	–
Percent unconcealed (“out”)	56.4; 1.19	55.4; 1.23	69.8; 3.90	0.0005	0.292
Psychological distress (Kessler)—mean (0–24)	8.82; 0.18	8.77 0.19	9.39; 0.78	0.4408	–
Percent severe psychological distress (SPD—Kessler >12)	26.2; 1.50	25.6; 1.54	33.9; 6.11	0.1903	–
How many days in the past 30 days was your mental health not good?	11.9; 0.34	11.9; 0.35	12.0; 1.38	0.9587	–

*Values shown are weighted for population and survey design. *N*, unweighted number of cases; SE, standard error; *P*, value of *p* of *t* test result; *d*, Cohen’s *D**.

Those who would later undergo SOCE experienced significantly higher ACEs in childhood. Of the eight ACEs measured, the SOCE alumni experienced an average of 4.2, almost one ACE higher than the 3.3 average for the non-SOCE group, although the corresponding effect size for this difference was marginal. Those who experienced SOCE were also much more likely to have been bullied in high school due to their sexual orientation. Thirty-eight percent of SOCE alumni, compared to only 18% of the non-SOCE group, had suffered frequent bullying in high school, a difference in effect of almost half a standard deviation. Lifetime sexual orientation victimization, a summary scale of eight adverse discrimination experiences, was also over twice as high for SOCE participants (3.5–1.3) as for other sexual minorities. This difference is highly significant, with a strong effect, at over 80% of a standard deviation. Forty-four percent of the SOCE alumni had been assaulted for their sexual orientation, compared to only 17% of those who had not participated in SOCE. The heightened adversity suffered by SOCE participants was not confined to the past. Significantly higher proportions of SOCE alumni had also experienced everyday discrimination, felt stigma and chronic strains, all measures of discrimination, stigma, and stress, within the past year.

Despite experiencing higher stress and stigma both currently and over their lifetimes, however, the SOCE alumni did not manifest higher psychosocial distress or even higher discomfort with an LGB sexual identity. There was no difference between the SOCE alumni and the non-SOCE group on internalized homophobia (as adjusted), psychological distress, the proportion found to be in severe psychological distress, and the number of days of poor mental health in the past month. Moreover, the SOCE alumni were more likely to be unconcealed (“out”) about their sexual minority identity. Almost half (44.6%) of sexual minorities who had not experienced SOCE concealed their sexual minority identity from family, friends, co-workers or healthcare providers, but such concealment dropped to under a third (30.2%) for those with SOCE experience. The proportion of those who were “out” to at least one constituency was almost 22 percentage points higher with SOCE experience, and over a quarter (25.6%) of SOCE alumni were completely unconcealed to any constituency, compared to only 18.2% of those without SOCE experience (not shown).

The absence of SOCE-associated internal distress or disability is mirrored by similarly benign findings regarding behavioral harm. Table 3 compares unadjusted mean values by SOCE experience for the seven behavioral harm outcomes measured by the Generations data: non-suicidal self-harm, substance abuse disorder, alcohol dependence, suicide ideation, suicidal intention, suicidal planning, and attempting suicide. None of the seven behaviors have any statistical association with having undergone SOCE therapy, as indicated by value of *p*s ranging from 0.30 to 0.92 for *t* tests testing the difference of means. Indeed, for five of the seven the population risk with SOCE therapy is identical, to two decimal places, to the risk with no SOCE, and the highest risk ratio for all seven behaviors is just 1.03. The current risk of these harm behaviors for those who have experienced SOCE is no different than it is for those who have not experienced SOCE. In sum, SOCE experience has no statistically discernible effect on the risk of any present harm measured in these data: psychological distress, lower mental health, alcohol dependence, substance abuse, suicide ideation, suicide planning, suicide intention, and attempting suicide.

**Table 3:** Unadjusted means and risk ratios, and adjusted AORs, showing current behavioral harm by experiencing SOCE, counts, and weighted proportions: probability sample of sexual minorities, United States, 2016–2018 (*N* = 1,518).

	Overall sample (*N* = 1,518), No (%; SE) or Mean (SE)	Experienced SOCE		Yes = No, *P*	Risk (odds) ratio	Multivariable AOR, *P*
		No (*N* = 1,410), No (%; SE) or Mean (SE)	Yes (*N* = 108), No (%; SE) or Mean (SE)			
**Behavioral harm outcomes**						
Self-harm (cutting, etc.)	47.23; 1.63	47.39; 1.69	45.08; 6.25	0.7217	1.00	1.03, 0.95
Substance use disorder (DUDIT)*—percent*	37.8; 1.58	37.8; 1.64	37.2; 5.85	0.9208	1.00	0.92, 0.80
Alcohol dependence (AUDIT-C)*—percent*	39.4; 1.55	39.1; 1.60	43.6; 6.15	0.4744	1.00	1.42, 0.23
Suicide ideation	45.4; 1.61	45.7; 1.66	42.5; 6.12	0.6167	1.00	0.76, 0.45
Suicide planning	32.9; 1.53	33.0; 1.58	31.7; 5.87	0.8341	1.00	0.71, 0.32
Suicide intention	15.6; 1.22	15.2; 1.25	21.0; 5.49	0.2984	1.02	1.24, 0.63
Suicide attempt	5.8; 0.84	5.8; 0.87	6.8; 3.28	0.7573	0.97	0.21, 0.03

*Values shown are weighted for population and survey design. *N*, number of unweighted cases; SE, standard error; *P*, value of *p* of *t* test result; AOR, adjusted odds ratio. Mean comparisons were unadjusted. Column 4 tests the difference of means; column 6 tests the departure of the AOR from unity. Multivariable logistic regression models for column 6 were weighted and adjusted for demographics (age, sex at birth, sexual identity, race/ethnicity, education, health, and income; see *Table 1*); minority stressors [ACEs, psychological distress (Kessler), past month mental health, concealed sexual identity, internalized homophobia (adjusted), bully victimization, lifetime victimization/discrimination, everyday discrimination, felt stigma, and chronic strains; see *Table 2*]*.

Observed equivalence in the population, of course, may reflect the net result of covariate influences that counteract that of SOCE therapy. The findings presented in Tables 1 and 2, showing lower socioeconomic status and higher minority stress among the SOCE alumni, suggest that such influences are more likely harm-enhancing than not. In this case equivalent harm would indicate that SOCE likely had an ameliorating effect to bring the predicted excess of harm due to minority stress and/or poor early life influences into equivalence with the non-SOCE population, who were subject to lower stress. It is possible, however, that the reverse is true, with SOCE aggravating unobserved influences that would have otherwise lowered current harm for the SOCE group below that of the non-SOCE group.

To address these possibilities, I examined logistic regression models adjusted for all of the demographic and stressor covariates presented in Tables 1 and 2. The corresponding adjusted odds ratios (AORs) for SOCE, with values of *p* indicating statistical significance, are reported in the Table 3 column labeled “Multivariable AOR.” Six of the seven AORs were non-significant, indicating that differences in stress and demography do not account for the lack of an association of SOCE therapy with these behaviors in the sexual minority population. Given this limitation, the sample AORs suggest that alcohol dependence and the declaration of suicide intentions may be moderately aggravated, and influences toward higher suicide ideation and planning may be moderately reduced, for the SOCE alumni.

The results for current suicide attempts are strikingly different. For this outcome, the AOR for SOCE is both statistically significant and strongly negative, indicating that those exposed to SOCE were about five times less likely to currently attempt suicide than were persons of comparable demographics and stress experience who were not exposed to SOCE. Unlike for the other six harm outcomes, the model for suicide attempts does not have an acceptable fit to the data, indicating that it is necessary to consider other factors, beyond the scope of the present study, to fully account for current suicide attempts among sexual minority persons. With regard to the question under examination here, however, the model indicates that the experience of SOCE therapy was associated with a sizeable decrease in the risk of a current suicide attempt that would otherwise be predicted by higher exposure to childhood and minority stress experienced by the SOCE alumni.

**Figure 1:**
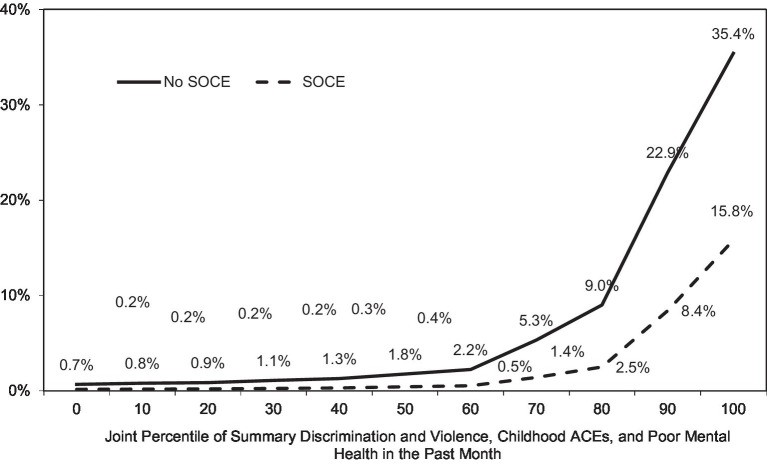
Predicted probability of current suicide attempt with increasing minority stress, adverse childhood experiences (ACEs) and poor mental health, by experience of sexual orientation change efforts (SOCE): probability sample of United States sexual minorities 2016–2018 (*n* = 1,518). *Source*: Generations Data, Wave 1, Williams Institute 2016 (*n* = 1,518). Shown are predicted outcomes from Table 3 suicide attempts model.

Figure 1 illustrates the effect. The figure reports the predicted probability of a suicide attempt by joint deciles of three stressor covariates: ACEs, lifetime sexual orientation victimization and discrimination, and current poor mental health. Two effects in the figure should be noted. First, the probability of suicide attempts increases with increasing stress, but the increase is not linear. The marginal effect of higher levels of stress increases much more rapidly after the 50th or 60th percentile. Like the straw that broke the camel’s back in the fable, a unit of additional stress on top of that of persons who were already highly stressed increased suicide attempt risk by a much greater amount than if it added to a low level of stress. Second, at every level of stress, past exposure to SOCE was associated with reduced suicide attempt risk, compared to those not exposed to SOCE. However, the effect of SOCE also increased sharply at higher levels of stress. In this model, for persons of equivalent stress, exposure to SOCE reduces the risk of suicide attempt, but we know from Table 2 that stress is not equivalent between the SOCE and the non-SOCE groups. In the joint distribution shown in Figure 1, median stress for the non-SOCE group is roughly 12 percentile points lower than that of the SOCE group. The figure illustrates that, as a result of these combined effects, the following claims can both be true: (a) SOCE reduces suicide attempt risk; and (b) the unadjusted probability of suicide attempts for SOCE alumni is equal to or greater than that of persons not exposed to SOCE.

It should be emphasized that this effect, while present in the data, does not by itself fully explain the observed equivalence of the SOCE and non-SOCE groups on the risk of current suicide attempts. The development of a comprehensive model of suicide attempts among sexual minorities awaits further research beyond the scope of the present study.

## Discussion

The present findings starkly contradict those of a body of research that claims to find invidious harm, in particular suicidal morbidity, among persons exposed to SOCE therapy. By contrast, the present study, examining a large, credible representative sample of sexual minority persons using multiple well-validated measures appropriately sensitive to harm, has found that the experience of SOCE therapy was unrelated to any measure of present harm. Those who had undergone SOCE were no more likely to experience psychological distress or poor mental health, to engage in substance or alcohol abuse, to intentionally harm themselves, or to think about, plan, intend or attempt suicide, than were those who had not undergone SOCE. This finding is all the more striking since the SOCE alumni examined were characterized by conditions that, in prior research, have repeatedly been associated with higher levels of such behaviors: an unsuccessful SOCE outcome, higher minority stress, higher childhood ACEs, and lower socioeconomic status. This group constituted not only a test, but a stress test of the hypothesis that SOCE therapy induces harm. If any group were likely to suffer harm from SOCE therapy, this group would have done so.

To the extent that minority stress may induce or encourage the risk of suicidal behaviors, furthermore, in these data it did so less strongly among persons exposed to SOCE than otherwise for the most serious suicidal behavior—suicide attempts. Although the reduction in suicide attempt risk with SOCE was sufficient to bring suicide attempts for the SOCE alumni into equivalence with non-SOCE group, with its lower experience of stress, this need not be the case. As Figure 1 illustrates, due to their higher exposure to stress and childhood adversity, it is possible that the SOCE alumni could benefit from SOCE exposure and still also have shown higher harm.

As with any therapeutic intervention, clinical concern for aggregate outcomes following SOCE is rightly focused not on history but on prognosis. It would be a perverse policy indeed, for example, for heart surgery to be discouraged or even banned because those undergoing it experienced higher rates of cardiac dysfunction than the general population before the surgery. Yet that is the form of argument being put forward in many settings to restrict or ban SOCE therapy for sexual minorities. As the present study has found, the majority of suicidal morbidity occurred prior to SOCE, and the most serious form of suicidal morbidity was reduced following SOCE. Yet Blosnich et al. (2021), for example, dismissed concerns that morbidity may have predated SOCE exposure in advocating a ban on SOCE due to its association with higher lifetime suicidal morbidity. Other recent studies have presented the same flawed form of argument (Ryan et al., 2020; Salway et al., 2020; del Río-González et al., 2021). For these reasons, proposals to restrict therapeutic interventions based on superficial claims of lifetime harm should be met with skeptical caution.

Proponents of the argument from lifetime suicidality have attempted to defend such dubious reasoning by claiming that persons undergoing SOCE are more exposed, as a group, to minority stressors, which in turn induce greater personal harm. Blosnich et al. (2020, p. 1027–1028), for example, attributed harm from SOCE to “the construct of perceived burdensomeness” resulting from minority stress, calling for further investigation into “specific constructs and mechanisms (e.g., enacted stigma, internalized stigma, and identity concealment) that could incite perceived burdensomeness and create the risk of suicidal thoughts and behaviors among survivors of SOCE.”

This argument also founders on the direction of causation. Since most suicidal morbidity occurred before recourse to SOCE, it is more likely that higher stress induced recourse to SOCE than the reverse, rendering this claim a good argument for reducing minority stress but a poor argument for restricting SOCE. The present findings, moreover, confirm predictions of higher minority stress, both currently and over their lifetimes, but fail to find any corresponding present harm, among those exposed to SOCE. Recently Meyer, the original proponent of MST, similarly found that changes in minority stress were not correlated with cohort trends in suicidal behavior in the Generations data (Meyer et al., 2021).

It is not possible for the present study to determine the mechanisms which reduce suicide attempts among former SOCE participants, but some suggestions in support of future research may be in order. With respect to MST, the present findings suggest three possibilities: either SOCE ameliorates the risk of harm deemed to be created by minority stress experiences; or those experiences do not induce harm, or as much harm, as MST predicts; or a combination of both.

Minority stress theory itself theorizes that minority stress may increase resilience under certain conditions (Meyer, 2003). In examining the possibilities, research might be rewarded by more fully elaborating the association between minority stress and harm, as in, for example, stress process theories which examine the interaction of individual characteristics with social processes, including cultural norms, in affecting stress responses throughout the life course (Pearlin et al., 1981; Lazarus and Folkman, 1984; Carver and Connor-Smith, 2010). In their classic typology of former SOCE participants, Shidlo and Schroeder found that about 10% of those they interviewed experienced a “resilient recovery of gay identity: …these participants reported few or no long-term damaging effects and actually felt strengthened by their experience of having tried to change. Their failure at conversion therapy freed them to embrace their gay or lesbian identity without ambivalence or guilt” (Shidlo and Schroeder, 2002, p. 254). It may be that this result is more common than was reflected in Shidlo and Schroeder’s non-probability sample, accounting for the results observed in the present study. These ideas are presented as suggestions to be sorted out by future research and not as firm conclusions from the present study.

Scholarly proponents of SOCE restrictions have also suggested that restrictions or bans are warranted, or perhaps acceptable on other grounds, because it is not effective (Blosnich et al., 2020; Ryan et al., 2020). As already noted, this claim is empirically dubious because the evidence cited for it is based almost exclusively on samples of self-identified sexual minorities, which have explicitly screened out anyone for whom SOCE may have been successful. But even when SOCE is unsuccessful, as must be true sometimes even if it is not true always, such a deficit would only be pertinent to a risk/benefit evaluation if there were a corresponding risk. The present study has found that, even for persons for whom SOCE has had no efficacy, there is no discernible psychosocial risk.

### Limitations

As Blosnich et al. (2020, p. 7) noted, the SOCE measure developed by the Generations researchers has not been validated and may not capture all forms of SOCE experience or all persons engaging in SOCE. They speculated that this may be due to greater concealment among SOCE alumni, but the present study has found the opposite to be true: SOCE alumni were significantly more likely to be “out” about their sexual orientation than were those not exposed to SOCE (See Table 2). Rather, the SOCE question likely was specified too narrowly: “Did you ever receive treatment from someone who tried to change your sexual orientation?” This prescriptive wording seems not to take seriously the claim of many SOCE practitioners that the goal of such therapy is to resolve psychological distress or value conflicts related to same-sex attractions, behavior or identification, which may or may not involve seeking to modify the current state of these elements of the client’s sexuality. (Opponents still define this as SOCE because the therapy is still open to the possibility of changing sexual orientation if desired.) Likely confusion or uncertainty over the SOCE question may be evidenced by the fact that almost half (49.1%, SE 6.12) of those who responded “Yes” to the SOCE question, indicating that they had gone through SOCE therapy, did not agree with the statement, “I have tried to stop being attracted to people who are the same sex as me.”

In addition to low specificity, the SOCE measure may also exhibit low sensitivity. In the Generations data, attempts to resolve or change same-sex attractions were much more widespread in the sexual minority population than was indicated by the proportion who have undergone SOCE therapy. Almost a third (30.9, 95% CI 27.9–33.9) of the sexual minority population reported having attempted to change their sexual orientation to become heterosexual, but the SOCE question only captured under 7% of respondents, an amount four times lower.

Although the Generations data used in the present study have many strengths, they also have some limitations. The strong complex survey design assures that they were accurately representative of the target population, but they did not include the entire LGB population. As with any cross-sectional data, causation cannot be attributed with certainty. Some age ranges were screened out, as were persons below a fifth-grade education and some smaller racial minority groups.

## Conclusion

Examining a strong representative sample of sexual minority persons in the United States, the present study has found that the prevalence of current or prospective behavioral harm among sexual minorities is statistically identical for those who have experienced SOCE and those who have not. These findings not only fail to find harm, but also present positive evidence of the absence of harm, from SOCE therapy. With respect to suicide attempts, SOCE appears to mitigate the harm attributable to elevated stress. Further study is needed to clarify the reasons for the absence of current harm, despite not only higher minority stress, but also higher childhood stress and lower socioeconomic status, experienced by those exposed to SOCE.

## Data Availability Statement

The data for this study are publicly available from the following data repository: Meyer, Ilan H. Generations: A Study of the Life and Health of LGB People in a Changing Society, United States, 2016-2019. Inter-university Consortium for Political and Social Research [distributor], 2020-08-25. https://doi.org/10.3886/ICPSR37166.v1.

## Ethics Statement

Ethical review and approval was not required for the current study in accordance with the local legislation and institutional requirements. As a secondary analysis of pre-existing public data, the present study’s methods were determined to be exempt from human subject ethical review under 45 CFR 46.104 by the Catholic University of America Institutional Review Board in Certificate 21-0016 issued March 12, 2021. The patients/participants provided their written informed consent to participate in the underlying survey data collection.

## Author Contributions

DS: conceptualization, data analysis, writing—original draft preparation, and writing—review and editing.

## Funding

This work was funded in part by The Catholic University of America, Research Grant 200230.

## Conflict of Interest

The author declares that the research was conducted in the absence of any commercial or financial relationships that could be construed as a potential conflict of interest.

## Publisher’s Note

All claims expressed in this article are solely those of the authors and do not necessarily represent those of their affiliated organizations, or those of the publisher, the editors and the reviewers. Any product that may be evaluated in this article, or claim that may be made by its manufacturer, is not guaranteed or endorsed by the publisher.
