# Corrigendum: Absence of Behavioral Harm Following Non-efficacious Sexual Orientation Change Efforts: A Retrospective Study of United States Sexual Minority Adults, 2016–2018

**DOI:** 10.3389/fpsyg.2022.906401

**Published:** 2022-04-26

**Authors:** D. Paul Sullins

**Affiliations:** ^1^Department of Sociology, The Catholic University of America, Washington, DC, United States; ^2^The Ruth Institute, Lake Charles, LA, United States

**Keywords:** sexual orientation change efforts, conversion therapy, minority stress, suicide, sexual orientation

In the original article, there was an error. The description of “current suicidality” stated that this was “indicated by having engaged in the behavior at least once in the past year” but failed to include that this was measured at both Wave I and the Wave 2 one-year follow-up. This information is necessary in order to replicate the findings.

A correction has been made to *Measures, Behavioral Harm, Suicidal Behavior*. The corrected paragraph is shown below.

Suicidal behavior was assessed using an instrument developed by the Unite States Army to assess risk in service members (Nock et al., 2014). Four questions asked, “Did you ever … in your life have thoughts of killing yourself?”; … have any intention to act on thoughts of wishing you were dead or trying to kill yourself?; … think about how you might kill yourself (e.g., taking pills, shooting yourself) or work out a plan of how to kill yourself?; … make a suicide attempt (i.e., purposefully hurt yourself with at least some intention to die)?” Response options were “No,” “Yes, once,” and “Yes, more than once.” Follow-up questions for the yes responses asked how old the respondent was when they engaged in the suicide behavior or both the first and most recent of multiple instances of that behavior. For each behavior, current suicidality was indicated by having engaged in the behavior at least once in the past year, as indicated at Wave 1 or Wave 2.

In the original article, there was also a mistake in Table 3 as published. The N for the overall sample and in the legend was incorrectly shown as 833. It should be 1,518. The corrected Table 3 appears below.

**Table 3:** Unadjusted means and risk ratios, and adjusted AORs, showing current behavioral harm by experiencing SOCE, counts, and weighted proportions: probability sample of sexual minorities, United States, 2016–2018 (*N* = 1,518).

		**Experienced SOCE**				
	**Overall sample (*N* = 1,518), No. (%; SE) or Mean (SE)**	**N,o (*N* = 1410), No. (%; SE) or Mean (SE)**	**Yes (*N* = 108), No. (%; SE) or Mean (SE)**	**Yes = No, *P***	**Risk (odds) ratio**	**Multivariable AOR, *P***
**Behavioral harm outcomes** Self-harm (cutting, etc.)	47.23; 1.63	47.39; 1.69	45.08; 6.25	0.7217	1.00	1.03, 0.95
Substance use disorder (DUDIT)–*percent*	37.8; 1.58	37.8; 1.64	37.2; 5.85	0.9208	1.00	0.92, 0.80
Alcohol dependence (AUDIT-C)–*percent*	39.4; 1.55	39.1; 1.60	43.6; 6.15	0.4744	1.00	1.42, 0.23
Suicide ideation	45.4; 1.61	45.7; 1.66	42.5; 6.12	0.6167	1.00	0.76, 0.45
Suicide planning	32.9; 1.53	33.0; 1.58	31.7; 5.87	0.8341	1.00	0.71, 0.32
Suicide intention	15.6; 1.22	15.2; 1.25	21.0; 5.49	0.2984	1.02	1.24, 0.63
Suicide attempt	5.8; 0.84	5.8; 0.87	6.8; 3.28	0.7573	0.97	0.21, 0.03

*Values shown are weighted for population and survey design. N, number of unweighted cases; SE, standard error; P, p-value of t-test result; AOR, adjusted odds ratio. Mean comparisons were unadjusted. Column 4 tests the difference of means; column 6 tests the departure of the AOR from unity. Multivariable logistic regression models for column 6 were weighted and adjusted for demographics (age, sex at birth, sexual identity, race/ethnicity, education, health and income; see Table 1); minority stressors [ACEs, psychological distress (Kessler), past month mental health, concealed sexual identity, internalized homophobia (adjusted), bully victimization, lifetime victimization/discrimination, everyday discrimination, felt stigma, and chronic strains; see Table 2]*.

The author apologizes for these errors and states that they do not change the scientific conclusions of the article in any way. The original article has been updated.

## Publisher's Note

All claims expressed in this article are solely those of the authors and do not necessarily represent those of their affiliated organizations, or those of the publisher, the editors and the reviewers. Any product that may be evaluated in this article, or claim that may be made by its manufacturer, is not guaranteed or endorsed by the publisher.

